# CRISPR/Cas9-Mediated Mutagenesis of the Granule-Bound Starch Synthase Gene in the Potato Variety Yukon Gold to Obtain Amylose-Free Starch in Tubers

**DOI:** 10.3390/ijms23094640

**Published:** 2022-04-22

**Authors:** Stephany Toinga-Villafuerte, Maria Isabel Vales, Joseph M. Awika, Keerti S. Rathore

**Affiliations:** 1 Department of Soil & Crop Sciences, Texas A&M University, College Station, TX 77843-2474, USA; stephanyetv2@tamu.edu (S.T.-V.); joseph.awika@ag.tamu.edu (J.M.A.); 2 Department of Horticultural Sciences, Texas A&M University, College Station, TX 77843-2133, USA; isabel.vales@ag.tamu.edu

**Keywords:** amylose, amylopectin, CRISPR/Cas9, gene knockout, GBSS, sgRNAs, rapid visco analyzer, *Solanum tuberosum*, targeted mutagenesis

## Abstract

Potato (*Solanum tuberosum* L.) is the third most important food crop after rice and wheat. Its tubers are a rich source of dietary carbohydrates in the form of starch, which has many industrial applications. Starch is composed of two polysaccharides, amylose and amylopectin, and their ratios determine different properties and functionalities. Potato varieties with higher amylopectin have many food processing and industrial applications. Using *Agrobacterium*-mediated transformation, we delivered Clustered regularly interspaced short palindromic repeats and CRISPR-associated protein 9 (CRISPR/Cas9) reagents to potato (variety Yukon Gold) cells to disrupt the granule-bound starch synthase (*gbssI*) gene with the aim of eliminating the amylose component of starch. Lugol-Iodine staining of the tubers showed a reduction or complete elimination of amylose in some of the edited events. These results were further confirmed by the perchloric acid and enzymatic methods. One event (T2-7) showed mutations in all four *gbss* alleles and total elimination of amylose from the tubers. Viscosity profiles of the tuber starch from six different knockout events were determined using a Rapid Visco Analyzer (RVA), and the values reflected the amylopectin/amylose ratio. Follow-up studies will focus on eliminating the CRISPR components from the events and on evaluating the potential of clones with various amylose/amylopectin ratios for food processing and other industrial applications.

## 1. Introduction

Potato (*Solanum tuberosum* L.) is the leading tuber crop and third most important food crop after rice and wheat. Potato originated in the Andean Highlands, where it was domesticated around 8000 years ago [1,2]. Following the introduction of potato in Europe by Spaniards, its adoption as food was slow at first, but then it took off over time in Europe and the rest of the world. Potatoes are now popular as food among the rich and poor alike in many developed and developing countries. As an energy-dense crop that yields four times more calories per hectare compared to the grain crops, it has come to be regarded as a food security crop in some countries [2] and has also acquired a reputation as a smallholder, low-tech crop. As a food item, it is cooked in different ways and is an important ingredient in a variety of dishes for immediate consumption, as well as being processed as frozen French fries, chips/crisps and other snacks for longer term use. In addition to its use as food, potato starch has many applications in processed food, paper, adhesive and textile industries [3]. Thus, potato represents an important target crop that can be modified either to improve its nutritional qualities or for industrial applications.

Starch is a semicrystalline polymer mainly composed of two types of polysaccharides: amylose and amylopectin. Depending on their ratio, the physical and chemical properties of the starch can vary, such as gelatinization, swelling capacity, solubility in water, texture and recrystallisation [4,5,6,7,8,9,10]. Amylose, a linear polymer composed of α-D-glucose units linked by α-(1,4)-bonds, constitutes 20 to 30% of the starch present in plants. It is produced from ADP-glucose as a result of enzymatic activity of granule-bound starch synthase (GBSS). In contrast, amylopectin is a polysaccharide composed of relatively short α-(1,4)-glucan chains that are joined together by α-(1,6)-bonds at branch points forming highly branched, higher molecular weight compounds that constitute 70 to 80% of the starch in most plants [6,10].

While higher amylose starches are currently used in products targeting health applications due to their lower glycemic index, starches higher in amylopectin have many processed food and other industrial applications due to their unique functional properties. Amylopectin gelatinizes easily to a clear, soft paste that is more stable and less prone to syneresis upon aging. Because of the shorter average chain length and extensive branching, amylopectin has a slower rate of retrogradation. As such, it is the preferred form of starch for use as stabilizer and thickener in food products and as an emulsifier in salad dressings [6]. Another important feature of higher amylopectin starch is its freeze–thaw stability. For example, frozen tapioca starch with higher amylopectin showed no loss of water upon thawing compared to other starch sources with lower amylopectin content [11]. Interestingly, waxy rice with virtually no amylose showed very little or no separation of water when subjected to six freeze–thaw cycles [12]. In many countries, the primary raw material for ethanol production is starch-rich cereals, mainly corn. Ethanol forms the basis for all alcoholic drinks and is also used in vast quantities as a fuel supplement in the transportation sector. The starch in waxy corn is largely formed by amylopectin and has been shown to be responsible for significantly higher starch–ethanol conversion efficiency compared to that of normal corn [13]. Thus, amylose-free potato can also serve as a raw material that yields higher levels of ethanol compared to normal potatoes.

The Clustered regularly interspaced short palindromic repeats and CRISPR-associated protein (CRISPR/Cas) system, which acts as an adaptive defense mechanism in bacteria and archaea against invading viruses, has been repurposed to generate a double stranded break (DSB) in a desired gene within the genomes of Eukaryotes in a precise and targeted manner. Non-homologous end joining (NHEJ), the predominant, but error-prone DNA repair mechanism, usually creates INDELS (insertions/deletions) at the break site, thus resulting in a mutation. The simplicity and efficiency of the CRISPR/Cas system has made it the most popular technology to generate precise mutations in the genome to study gene function and also to create useful traits in crop plants [14,15,16].

The CRISPR/Cas9 system was used to generate amylose-free potato, and this was achieved using the protoplast system [17,18,19]. While the protoplast system has the advantage in that it makes it possible to directly obtain edited events that are devoid of any transgenes, the system is inefficient, expensive and prone to somaclonal variations, resulting in abnormal plants because of the longer time-frame required for regeneration [20]. The *Agrobacterium*-mediated method offers a simpler, less expensive and relatively quicker means to introduce CRISPR components to generate targeted mutations in plants. This system was used by Kusano et al. [21] to target the GBSS alleles in potato (cv. Sayaka) to eliminate amylose. Starting in 2017, we also employed this widely used *Agrobacterium* system to knock out the *gbss* gene in the popular yellow potato variety Yukon Gold strain TXYG79. The specific objectives were: (i) to evaluate whether it is possible to knock out all four alleles of a native gene from stably integrated CRISPR reagents, (ii) to examine whether the shoot organogenesis mode of regeneration in potato results in chimeric plants and tubers, (iii) to obtain edited events with tubers devoid of amylose. Results of this investigation are discussed in relation to previously published studies.

## 2. Results

**Figure 1:**
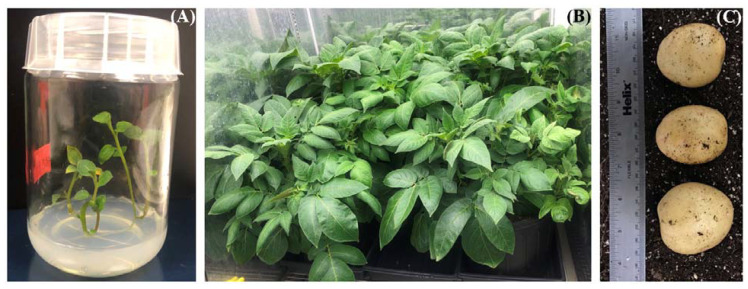
Regenerated, mutated Yukon Gold potato events. (**A**) Regenerated plantlets growing in vitro following *nptII*/Kanamycin-based selection. (**B**) Regenerated plants grown in soil in a growth chamber for tuber production. (**C**) Tubers collected two weeks after vine kill.

In vitro-grown TXYG79 plants were used to knock out the *gbss* gene in its tetraploid genome. An earlier study in our laboratory indicated that the CRISPR/Cas9 system was capable of mutating all four copies of the green fluorescent protein (*gfp*) gene in the potato genome [22]. Selection of events was based on the *nptII*/Kanamycin-based selection system as it resulted in higher transformant recovery. The regenerated potato events grew normally in vitro and also in soil under growth chamber conditions to produce tubers (Figure 1). We did not observe any obvious differences in terms of either yield or morphology (skin color, shape and size) of the tubers when comparing the edited events and the wild-type (WT).

### 2.1. Qualitative Histochemical Analysis Showed Various Phenotypes in the Knockout Events

**Figure 2:**
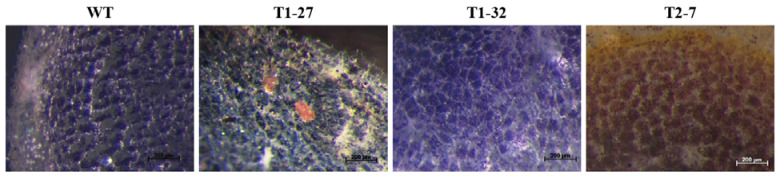
Examples of the different phenotypes detected via the histochemical Lugol-Iodine staining of microtuber sections. **WT**: wild-type (Yukon Gold, strain TXYG79) tuber showing typical blue staining of starch indicative of normal amylose/amylopectin ratio; **T1-27**: wherein Target site 1 was targeted for mutation, showing small sectors of complete knockout phenotype surrounded by largely blue-colored tuber section; **T1-32**: wherein Target site 1 was targeted for mutation, but does not show the knockout phenotype; **T2-7**: wherein Target site 2 was targeted for mutation showing complete knockout phenotype, i.e., reddish-brown-colored staining throughout the tuber section due to the absence of amylose.

**Figure 3:**
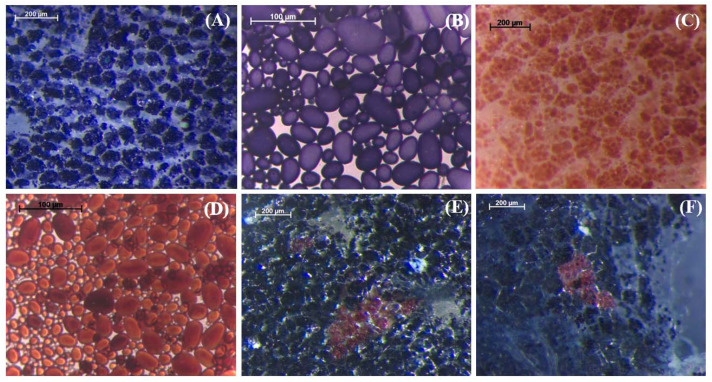
Histochemical Lugol-Iodine staining of sections from tubers produced by soil-grown plants showing different phenotypes. (**A**) wild-type (Yukon Gold, strain TXYG79) tuber section showing typical blue staining of starch indicative of normal amylose:amylopectin ratio; (**B**) stained starch granules from wild-type tuber; (**C**) tuber section from event T2-7 wherein Target site 2 was targeted for mutation showing a complete knockout phenotype (i.e., reddish-brown-colored staining due to the absence of amylose); (**D**) stained starch granules from event T2-7; (**E**) tuber section from event T1-27, wherein Target site 1 was targeted for mutation showing a small sector of complete knockout phenotype surrounded by largely blue-colored tuber section; (**F**) tuber section from event T2-2, wherein Target site 2 was targeted for mutation showing a small sector of complete knockout phenotype surrounded by largely blue-colored tuber section.

Microtuber sections from each event and in vitro-grown wild-type (WT) control plants were examined histochemically using the Lugol-Iodine method (Figure 2). For gRNA1, 37 individual events were evaluated, but none showed the expected complete knockout phenotype resulting from the absence of amylose (i.e., red-colored stained starch in the entire tuber), indicating that one or more alleles remained unmutated. However, one event (T1-27) showed a few small, red-colored patches randomly distributed throughout the mostly blue-colored microtuber section (Figure 2). Three Target 1 events (T1-1, T1-27 and T1-32) were grown in soil in a growth chamber for more extensive characterization. Tubers from soil-grown event T1-27 also showed red-colored patches, confirming the results seen in microtubers (Figure 3).

For gRNA2, microtubers from 32 individual events were examined following starch staining. Only one event (T2-7) showed the red-colored, starch staining phenotype throughout the microtuber section (Figure 2), indicating absence of amylose in the tuber, and therefore complete knockout phenotype resulting from mutations in all four *gbss* alleles present in the tetraploid potato genome. Staining of tuber sections from a soil-grown plant of this event further confirmed these results (Figure 3). Tubers from two other events (T2-2 and T2-8) showed a few red-colored patches within a largely blue-colored tuber section (Figure 3; bottom panel). These three Target 2 events (T2-2, T2-7 and T2-8) were selected for more extensive characterization.

### 2.2. Molecular Characterization of Events Showed a Variety and Extent of Mutations

**Figure 4:**
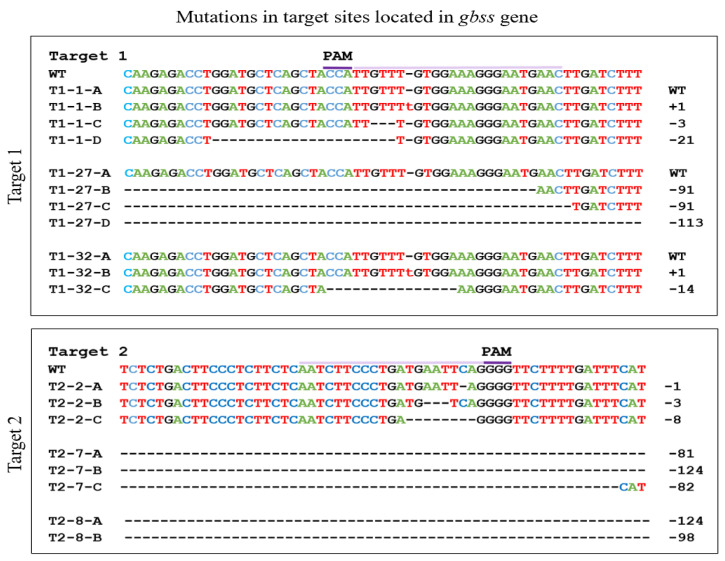
Nature of mutations ascertained by Sanger sequencing of clones obtained from PCR products that were amplified from the genomic DNA of leaves from three events per target site that were characterized extensively. Overlined: target sequence in the native *gbss* gene; dark purple line: PAM (protospacer adjacent motif) sequence; WT: wild-type sequence; T#-#-alphabet: the target number, the knockout event number for each target and the type of mutation obtained.

Indels (insertions or deletions) were seen in each event, with deletions being the most frequent type of mutation (Figure 4). However, two events had one bp insertion when Target site 1 was targeted for mutation (events T1-1 and T1-32). In each of the three events selected for Target 1, at least one allele was still intact. As reported earlier, even with one unmutated, functional allele of the *gbss* gene, there is little reduction in the levels of amylose in the potato tuber [17]. This would explain the largely blue-colored staining of the starch in these three events (Figure 2, Figure 3 and Figure 4).

When gRNA2 was used to generate mutations, we observed a clear knockout phenotype (reddish/brown staining, waxy phenotype) in one event, T2-7 (Figure 2, Figure 3 and Figure 4). The fact that this event showed an amylose-free phenotype but only three types of mutations suggests that two alleles likely had homozygous mutations (Figure 4). For the other two events where Target site 2 was subjected to mutation, event T2-2 showed three types of mutations, whereas event T2-8 showed only two types of mutations. While the sequencing results showing a lack of wild-type sequence would suggest that these events had one or two homozygous, biallelic mutations, these events did not show the phenotype of a complete knockout event. An explanation for this discrepancy is presented in the Discussion.

### 2.3. Quantitative Analysis of Starch Composition in Knockout Events Confirmed the Results of Histochemical Analysis

Specific gravity measurements (Table 1) allowed us to compare tubers from each event with the unmodified WT control tubers and thus determine if the genetic modification altered the dry matter content of the tubers. The complete knockout event (T2-7) was not significantly different from the WT control, with specific gravity values of 1.094 and 1.105, respectively. However, tubers from the event T1-27 with some reduction in amylose content and T1-32 with no reduction in amylose content had significantly lower specific gravity values of 1.082 and 1.063, respectively, compared to WT. The percentage of dry matter was calculated for tubers from each event and the values are also presented in Table 1. Only event T1-32 had significantly lower dry matter compared to WT.

**Figure 5:**
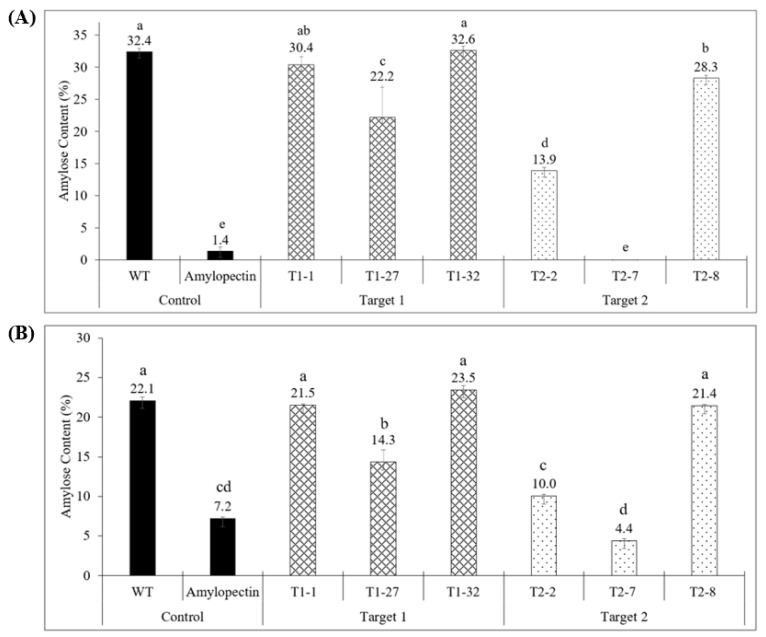
Amylose values in wild-type (WT, Yukon Gold strain TXYG79) and six different knockout events. (**A**) Colorimetric determination of amylose content using the perchloric acid method. (**B**) Colorimetric determination of amylose content using the commercial amylose/amylopectin Megazyme kit. A 100% amylopectin solution was also used as a control. Data represent mean ± SE; *n* = 4–6; different lower-case letters over bars indicate significant difference between plant types; *p* ≤ 0.05 (Student’s *t*-test).

The perchloric acid method for starch analysis showed that the WT control tubers had an amylose content of 32.4%. Tubers from events T1-1 and T1-32 (gRNA1) and event T2-8 (gRNA2) had percentages of amylose content not significantly different from that of the WT tubers. T2-7 (gRNA2), the complete knockout event, showed an absence of amylose in the tubers. Event T1-27 (gRNA1) and event T2-2 (gRNA2) at 22.2% and 13.9% amylose, respectively, showed lower amylose content in the tubers although not enough to be considered complete knockout events when compared to the WT (Figure 5A). As explained in the Discussion, the chimeric nature of these edited events can account for such results.

The Megazyme kit provided somewhat similar results as those obtained using the perchloric acid method. With this method, the WT control tubers gave an amylose content value of 22.1%. However, even a solution of pure amylopectin without any amylose showed a value of 7.2% (Figure 5B). Events T1-1 and T1-32 (gRNA1) and event T2-8 (gRNA2) showed percent amylose values not significantly different from that of WT tubers at 21.5%, 23.5% and 21.4%, respectively. The knockout event T2-7 showed the lowest percent amylose value of 4.4% in the tuber, which is even lower than the solution containing only amylopectin (7.2%). Event T1-27 (14.3% amylose) and event T2-2 (10% amylose) also showed significantly lower percentage amylose content in the tubers compared to WT, although not enough to be considered complete knockout events. Thus, while percent amylose content values are slightly different with the commercial kit, the overall trend is similar to that obtained with the perchloric acid methodology (Figure 5).

### 2.4. Viscosity Measurements in the Tuber Samples Confirmed Lack of Amylose in Event T2-7

**Figure 6:**
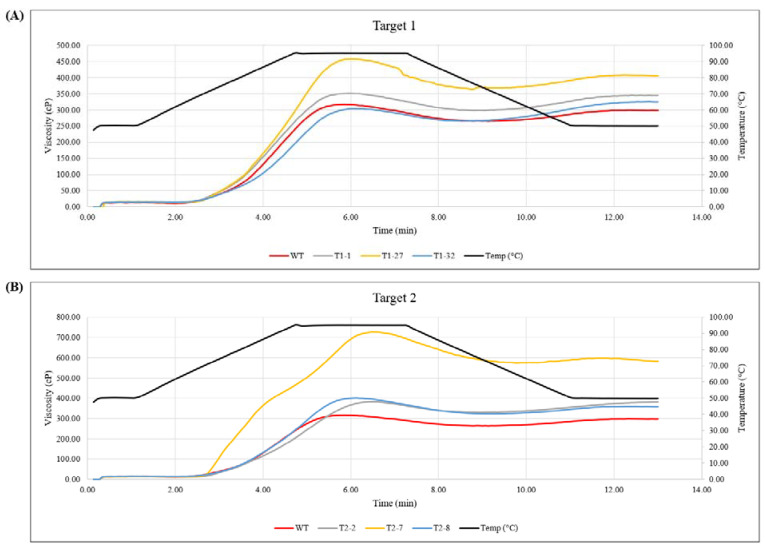
Viscosity and temperature profiles as determined by Rapid Visco Analyzer (RVA). (**A**) RVA profiles for control (unedited, wild-type (WT)) and edited events for Target 1. (**B**) RVA profiles for control (unedited, wild-type (WT)) and edited events for Target 2.

Viscosity was measured (in relation to temperature profile) using a Rapid Visco Analyzer (RVA; PerkinElmer). The same three events for each target (T1-1, T1-27 and T1-32, and T2-2, T2-7 and T2-8) were used for RVA analysis. The readouts from the RVA include viscosity profiles in relation to temperature profile for each sample. The complete knockout event, T2-7, showed a significantly higher peak (726 centipoise (cP)) and final viscosity (581 cP) as compared to the control (317 cP and 297.3 cP, respectively) and rest of the samples (Figure 6 and Table 2). The higher peak viscosity (PV) of the T2-7 indicates higher starch granule swelling capacity during cooking, an important functional property of waxy starches [23]. Furthermore, the higher final viscosity of the T2-7 indicates better cold paste viscosity, which suggests better functionality of the starch from complete knockout event as a thickener. Viscosity values for starches with higher levels of amylopectin are expected to be greater, thus the RVA results of various samples from the edited events bear this out (Figure 6 and Table 2). These results again confirm those obtained from histochemical and biochemical assays.

RVA profiles (Figure 6 and Table 2) also revealed subtle differences in the pasting properties of the different starch samples. These properties depend on the amylose/amylopectin ratio in the starch sample and informs the best use for a given type of starch. The breakdown value (which indicates paste stability [24]) for event T2-7 (153) was significantly higher compared to the WT value (53) (Table 2).

The setback viscosity is a measure of the gelling ability or retrogradation ability of starches [24] and indicates recrystallization of amylose molecules in the gel. This value for event T2-7 was 8 while the control was at 33.3, and the rest of the edited events showed values higher than 30 (Table 2). Again, this parameter reflects the lack of amylose in event T2-7. Although the pasting temperatures were generally not different among the treatments, the T2-7 showed a more rapid swelling during the cooking cycle than the rest of the samples (Figure 6). These characteristics of the starch help in maintaining food quality and stability during freeze–thaw cycles [25].

## 3. Discussion

The introduction of new traits in crops has relied on conventional breeding based on crosses with wild relatives or mutants created via radiation or chemical mutagenesis. The resulting progeny is selected based on incorporation of the desired new trait together with high yield and quality for a specific market class. Traditional breeding has disadvantages, such as the length of time it takes to establish the desired traits and the lack of control over undesirable traits brought in by linkage drag if a crop’s wild relatives or unadapted clones are used as parents [26]. Commercial potato is generally an autotetraploid, highly heterozygous and mainly propagated vegetatively (clonal crop). Currently, the varieties available in the market are the result of extensive breeding. Even though wild relatives with desirable traits are available, their incorporation into commercial varieties is a long and arduous process. Wild relatives could have different ploidy levels with different endosperm balance number (EBN) that creates crossing incompatibilities. Self-incompatibility and inbreeding depression in most potatoes, due to fixation of unfavorable alleles, further limits breeding efforts and the ability to incorporate novel traits [2,27].

Technologies exploiting the use of site-directed nucleases have been shown to be more precise and efficient in introducing new traits into crops, the CRISPR/Cas9 system being the most recent gene editing technology that has been widely adopted because of its ease of use. This system, which functions as an adaptive immune mechanism in bacteria, has become a popular tool to introduce mutations/modifications in the genomes of Eukaryotes. The Cas9 endonuclease, guided by a customized RNA, generates double stranded breaks (DSB) at the target DNA sequence in the genome [15,28,29]. The non-homologous end joining (NHEJ) repair of these DSBs usually results in mutations leading to loss of gene function (knockout) [14,15,30,31]. One of the appeals of gene editing is lower regulatory burden for the approval of modified crops compared to those created using traditional genetic modifications [29,31,32].

The *Agrobacterium* system offers an efficient and inexpensive means to introduce/alter genetic material in potato [33]. Previous studies have shown internodes as useful explants for *Agrobacterium*-mediated transformation; however, genotype dependence remains a limitation for the recovery of transformants [34]. In this study, *Agrobacterium*-mediated transformation of internode explants was used to introduce the CRISPR reagents in potato (Yukon Gold strain TXYG79) to knock out the *gbss* gene to eliminate amylose from the tubers.

The mode of regeneration and the length of time needed to recover edited events likely determine the presence of chimeric tissue in the regenerated plant. For example, in the case of cotton, the predominant mode of regeneration is via somatic embryogenesis, and the somatic embryos arise from undifferentiated callus after a long culture period, typically taking eight to twelve months [35]. This allowed Cas9 enough time to mutate the two transgene (*gfp*) alleles in most callus cells [30]. Thus, the somatic embryo originating from such a cell is likely to have all intended targets mutated in all of its cells. In the current study on potato, the mode of regeneration is via shoot organogenesis that also required considerably less time to recover plants. In addition, such adventitious shoots can be of multicellular origin [36]. Thus, de novo shoot organogenesis is likely to generate a genetically heterogeneous plantlet that contains cells with both mutated and unmutated alleles. Even the various mutated cells may differ in the type of mutations they carry [37]. All these factors offer a likely explanation for the discrepancy observed between the molecular results and the phenotype in some of the *gbss* knockout events (described below).

Sequence analyses were performed to determine the nature of mutations present in selected *gbss* knockout events. Four different types of variations would indicate different mutations in all four alleles, while a smaller number of mutations without the presence of an unmutated sequence would indicate the presence of homozygous mutations. Three events (T1-1, T1-27 and T1-32), wherein Target 1 was subjected to mutation, showed at least one wild-type copy of the *gbss* gene. As observed by Andersson et al. [17], even with a single functional copy of the *gbss* gene, the tuber produces a significant amount of amylose, thus retaining the blue color of the starch with the Lugol-Iodine staining. In agreement with this study, tubers from T1-1 and T1-32 showed blue-colored staining. A tuber from event T1-27 stained largely blue but showed some red-stained patches, indicating that the fourth *gbss* allele had undergone mutation in these tuber cells (Figure 3). The CRISPR components integrated in the genome of a plant remained active, and it is possible that these red-stained cells arose as result of late mutations in some of the tuber cells. When Target 2 was subjected to mutation, two to three types of mutations were observed in each of the three different events. None of these events had a wild-type, unmutated copy of the *gbss* gene. Only event T2-7 showed a complete lack of amylose and the red-stained starch phenotype. Absence of a wild-type copy and the fact that only three types of mutations are seen in this event suggest that it contains one biallelic, homozygous mutation. Thus, this event shows an exact match between the genotype and phenotype. Tubers from event T2-8 showed blue-colored staining throughout. T2-2 tubers were stained largely blue with a few red-colored patches (Figure 3). No wild-type copy of the *gbss* gene was observed in these two events, suggesting that all four alleles in their leaf cells were mutated (note that genomic DNA to conduct molecular analysis was obtained from leaves). However, the tubers that arose at the base of the stem may still carry one unmutated copy of the *gbss* gene, responsible for blue staining in the tuber. The chimeric nature of the potato regenerant and continued activity of the Cas9 to target the unmutated copy is evident from the tuber staining results for event T2-2 that showed a few red-stained patches surrounded by largely blue-stained starch. Such persistent activity of the genome-integrated CRISPR/Cas reagents resulting in a chimeric plant (in terms of different types of mutations in different sectors of the plant) has been shown in tetraploid cotton [38], *Medicago truncatula* [37] and *Arabidopsis* [39].

The levels of amylose in the mutated events were also measured by a perchloric acid method and an enzymatic method (Megazyme). This allowed us to correlate percentage of amylose present in the tuber with the histochemical data. Events that showed a blue-colored phenotype had a percentage of amylose similar to the WT (32.4% and 22.1% based on the perchloric acid and Megazyme kit methods, respectively). However, one event (T2-7) that showed a knockout, red-stained starch phenotype, completely lacked amylose in the tuber (0% and 4.4% based on the perchloric acid and Megazyme kit methods, respectively). Interestingly, the two events (T1-27 and T2-2) that had some red-colored patches surrounded by largely blue-colored staining in the tuber sections had significantly lower percentages of amylose 22.2% and 13.9% (perchloric acid method), respectively, compared to WT. This could be attributed to the presence of a proportion of tuber cells that had all four alleles of the *gbss* gene knocked out. Future greenhouse trials will involve determination of total starch content in the tubers of the various targeted events in comparison to the wild-type tubers and ascertain whether reduction/elimination of amylose results in net increase in amylopectin levels.

Specific gravity values represent indirect measure of the dry matter, i.e., it indicates the level of solids compared to the amount of water present in the tubers, with higher values being more desirable for processing purposes [40,41]. Events T1-27 and T1-32 showed significantly lower specific gravity values. However, no clear correlation was observed between amylose reduction and specific gravity, as indicated by the fact that tubers from the complete knockout event, T2-7, with no measurable amylose, had specific gravity value similar to the tubers from the non-edited plants. Specific gravity values for wild-type and various CRISPR events obtained in this investigation are within the range of those reported for Yukon Gold grown under field conditions (https://www.ars.usda.gov/ARSUSERFILES/20500500/POTATOREPORTS/2020/2020%20WESTERN%20REGIONAL%20RED%20REPORT.PDF (accessed 15 April 2022)). These results suggest that the relative levels of amylose and amylopectin do not affect the dry matter content of the tuber.

Viscosity profiles were determined with the aid of RVA in the *gbss* knockout events. Disruption of all four copies of the *gbss* gene is expected to eliminate the amylose component from the starch. Since the physicochemical properties of amylose and amylopectin are different, an alteration in the level of one kind confers different characteristics to the starch, including swelling, pasting and gelling properties that will determine its use in the industry [42].

The decrease or elimination of amylose content is expected to increase the PV. This can be seen clearly in event T2-7 that had a PV of 726 cP compared to WT with a PV of 317 cP. Such differences based on starch composition have been reported by other investigators. In cassava, Zhao et al. [43] showed that wild-type control had a PV of 885 cP, while the waxy transgenic event B9 had a value of 1094 cP. In another study comparing mutant waxy cassava with the wild-type, Toae et al. [44] observed the same trend. Similarly, in cereals such as maize, Sánchez et al. [45] found that the wild-type control had a PV of 176 cP, while the waxy variety had a PV value of 973 cP.

The setback value largely depends on the level of amylose present in the starch. This value relates to the cooling stage (or setback) during which retrogradation, or realignment of amylose and linear parts of amylopectin, occurs. The setback value is greater with higher levels of amylose and vice versa. In the current study, this value for the WT was 33.3 cP, while it was 8 cP for the complete knockout event T2-7. Such a correlation was also observed in a rice study where waxy varieties were analyzed [46].

The breakdown value of the starch is higher with a reduction in the amylose content. This value in the WT was 53 cP, while it was 98.3 cP for event T1-27 and 153 cP for the complete knockout event T2-7. Such a correlation was also observed in cassava [43,44] and in potato [45], where the waxy phenotype showed a higher breakdown value.

In the current study, pasting temperature values did not differ among the mutated events and the WT. This value for WT was 67 °C and for the complete knockout event T2-7 it was 69.1 °C. In a study by Sánchez et al. [45] that compared a waxy potato with WT potato, the pasting temperatures were 65.9 °C and 65.2 °C, respectively. The cassava investigation by Toae et al. [44] also showed a lack of difference in the pasting temperatures among different mutant events and the wild-type.

The evidence presented above demonstrates that the CRISPR/Cas technology could potentially be used to fine-tune the functionality of potato starch to meet broad applications. This is especially important given the growing consumer preference for foods that are not highly processed and chemically modified [47].

As mentioned earlier, the protoplast system has been used by Andersson et al. [17,18] and Johansen et al. [19] to eliminate amylose starch in potato. The main benefit of the protoplast system is that it allows direct delivery of the two required reagents: Cas9 enzyme and sgRNA (ribonucleoproteins, RNP). It is also possible to deliver RNP via gene gun or biolistic bombardment. This involves the use of gold or tungsten particles coated with RNPs for delivery to cells [48]. The major strength of these alternative methods is the avoidance of transgene integration in the cellular genome, which reduces the regulatory burden in some countries and allows the edited plants to be labeled as “GMO-free”. However, in the study conducted by Andersson et al. [17], using the potato variety Kuras, 25% of the regenerated plants were stunted and eventually died. They attributed this to the accumulation of somaclonal variations during the long culture period needed to regenerate plants following protoplast transformation. Other weaknesses of the protoplast transformation method are the requirement for expensive enzymes and complexity of the entire isolation and regeneration procedures. Thus, the edited plants derived from this method are more likely to contain somaclonal variations due to much longer culture periods. Such genetic variations are common when using protoplasts for potato transformation [20]. However, in comparison to the protoplast method, the *Agrobacterium* system is less expensive, as it does not require costly chemicals. In addition, the *Agrobacterium* method allows delivery of CRISPR reagents to cells within an organ, such as a leaf, followed by regeneration via direct organogenesis. The shorter time frame to obtain the regenerants reduces the problems created due to somaclonal variations. Thus, for the study conducted by Kusano et al. [21] and in the current investigation, the *Agrobacterium* system was used to deliver the CRISPR reagents. Another advantage of this method is that stable transgenic activities of Cas9-sgRNA continue until all the targets are mutated. Conversely, the possibility exists that continued activity of the CRISPR system causes off-target mutations. Efforts are underway in several laboratories to develop Cas9 variants with greater capabilities to discriminate against mismatches between the guide RNA and target DNA sequence. A recently developed variant (SuperFi-Cas9) reduces off-target DNA cleavage while maintaining its efficiency to generate DSBs at the target site [49].

In this study, we demonstrated the ability of the CRISPR/Cas9 system to generate mutations within the genome of a commercial variety of potato, Yukon Gold strain TXYG79. The native gene, *gbss*, was targeted for mutations in order to eliminate amylose in the starch of the tubers. Sequence analyses of the mutations showed insertions, deletions, homozygous mutations, biallelic mutations and also unmutated copies. Shoot organogenesis, as the mode of regeneration in potato, likely results in a chimeric T0 plant. This fact became evident from the results (both histochemical assays and starch composition analyses) obtained for the *gbss* knockout events (T1-27 and T2-2). A single complete knockout event (T2-7) obtained in this investigation shows that while the shoot organogenesis mode of regeneration complicates the use of the CRISPR system in potato, it is possible to obtain a complete knockout of a target gene in this tetraploid species.

The amylose-free knockout event, T2-7, should find industrial applications in the future in traditional sectors such as paper, adhesive, textile, bioplastic and ethanol industries. Tuber starch from this event, because of its freeze–thaw stability without the need for chemical modifications, should be useful for frozen food production. As mentioned earlier, bioethanol yields are higher when amylopectin serves as the source of starch, whether the conventional, thermal process is used to gelatinize the starch prior to hydrolysis and fermentation or the raw-starch hydrolysis of the cold fermentation is used [13,50]. Thus, potatoes with amylopectin as the exclusive form of starch should also yield more ethanol for industrial use or to create alcoholic beverages. Several new transgenic traits in potato have been deregulated in the U.S. in the last seven years [51,52]. While there is reluctance in countries outside the U.S., including some Latin American countries, in the public acceptance of edible GM crops such as potato, it is less likely to generate the same degree of opposition if the product derived from tubers is used strictly for industrial purposes. CRISPR components still present in the knockout events could be removed by selfing and selecting progeny that are free from the T-DNA but still maintain the desired amylose-free phenotype. Later, follow-up field trials will be conducted to assess agronomic and quality parameters of the knockout events in comparison with the unedited potatoes (Yukon Gold strain TXYG79), and industrial applications of amylose-free potatoes will be explored.

## 4. Materials and Methods

### 4.1. Plant Material and Binary Vector

Yukon Gold strain TXYG79 was used to introduce the Cas9-gRNA construct to knock out the *gbss* gene. Yukon Gold is a fresh market, cultivated potato variety with early- to mid-season maturity, slightly oval tubers, yellow skin, yellow flesh and medium-high specific gravity (IANR, [53]). TXYG79 was selected by the Texas A&M University Potato Breeding Program based on reduced number of eyes on tubers. The binary vector was assembled using plasmids pTC212 (containing Cas9 gene that was codon optimized for Arabidopsis [54]), pTC241 (used to assemble specific sgRNA expression cassette) and pCGS752 (base binary vector), provided by Dr. Daniel Voytas, University of Minnesota. To design gRNAs, the sequence of the target gene, *gbss*, was obtained from the database Spud DB-Potato Genomics Resource [55]. Primers were designed based on this sequence to PCR-amplify the *gbss* gene from Yukon Gold strain TXYG79. The sequence of the amplicon matched that of the *gbss* gene in the database. Two different target sites were identified with the aid of web-based tools: sgRNA Scorer 2.0 [56], WU-CRSPR [57] and CRISPR-P 2.0 [58]. Two separate sets of oligonucleotides were synthesized corresponding to the two target sites (Target1-For: aaacTTGTTTGTGGAAAGGGAATG; Target1-Rev: gattgCATTCCCTTTCCACAAACAA and Target2-For: gattgAATCTTCCTGATGAATTCAG; Target2-Rev: aaacCTGAATTCATCAGGAAGATT). Each pair was annealed, phosphorylated and introduced into pTC241. This pTC241 and pTC212 were then cloned into the binary vector pCGS752 by Golden Gate cloning method using AarI restriction enzyme (Thermo Fisher Scientific, Waltham, MA, USA) [28,59]. Each of the constructs was transferred separately into *Agrobacterium tumefaciens* (LBA4404) cells. The transformation methodology used was essentially that described by Chetty et al. [60].

### 4.2. Qualitative Analysis of Knockout Events

The expected phenotype could be examined more easily at the tuberization stage, hence all the regenerated, independent events obtained following transformation were grown in vitro until microtubers developed. The microtubers growing on each individual event were tested via the histochemical Lugol-Iodine (10% of KI and 5% of I in distilled water) staining method. The expected knockout, lower amylose phenotype, was visible as red-colored starch staining instead of the normal blue coloration obtained from wild-type tubers that also contain amylose starch.

Three individual events for each target, with and without the desired phenotype, were moved to soil (Pro-Mix BX Mycorrhizae) and grown in a growth chamber (temperature: 18 °C; photoperiod: 16 h light/ 8 h dark; fertilizer: Miracle-Gro once a week) to obtain plants that produce normal-sized tubers. These tubers were hand sectioned and also tested via the histochemical Lugol-Iodine method to further confirm the phenotype seen previously in the microtubers. Once the qualitative results from the tubers grown in soil were obtained, the remaining tubers (specifically the eyes) were used to generate additional clones that were then used for the molecular characterization and quantitative starch composition/property analyses.

### 4.3. Molecular Characterization of Mutations

To ascertain the nature of mutations present in the knockout events, PCR primers (Target 1, located in Exon 1: Forward-ATGGCAAGCATCACAGCTTCAC, Reverse-CATTGTCCAGATAATCTAGTCCAGC; Target 2, located in Exon 7: Forward-TCACTCGATTGCACGTTACC, Reverse-GTAAAGGTTTTGCGTCCATGACC) were designed flanking each of the target sites. Amplicons were then cloned into pMiniT^TM^ vector using a PCR Cloning kit (New England Biolabs, Ipswich, MA, USA) followed by its transformation into DH5α *E. coli*. At least twenty bacterial colonies for each target were used to extract DNA that were then sequenced via Sanger sequencing. These sequences were aligned to the original gene sequence that allowed us to determine the nature of mutations.

### 4.4. Specific Gravity Measurements and Quantitative Analysis of Starch Composition in the Putative Knockout Events

The harvested tubers from each soil-grown plant were first weighed in the air and then in water in order to calculate specific gravity values for each event using the equation: Specific gravity = (Weight in air)/(Weight in air—Weight in water). The tubers were then freeze-dried following the method described by Fajardo et al. [61]. This involved cutting the tubers into small cubes, storing them in a freezer overnight at −80 °C, and then subjecting them to freeze drying. After five days of freeze drying (LABCONCO, FreeZone console freeze dryer 6L −50 °C Series, Kansas City, MO, USA), the samples were ground and stored at −80 °C. The percentage of dry matter content in the tubers from various events was also calculated by obtaining the weight of the samples before and after freeze drying.

Two different methodologies were used to measure the percentage of amylose present in the tubers from each event. The first method used was perchloric acid [61] and the second one was based on an enzymatic reaction (Megazyme—Amylose/Amylopectin Assay Kit). For the perchloric acid method, first an amylose/amylopectin standard curve was generated using different proportions of each compound. From each biological sample, 20–30 mg of freeze-dried material was mixed with 500 µL of 45% (*w*/*v*) perchloric acid solution in a 50 mL plastic tube and incubated at room temperature for four minutes. Then, 16 mL of ultra-pure water was added, and the contents were mixed by vortexing. Following incubation at room temperature for ten minutes, 40 µL supernatant was transferred to a well of a microtiter plate. After adding 50 µL of iodine solution (2 g KI and 1 g I_2_ dissolved in 1 L of ultra-pure water) to each well, the absorbance was read at 550 nm and 620 nm on SpectraMax^®^ 190 Absorbance Plate Reader (Molecular Devices, San Jose, CA, USA). Percentage of amylose in the sample was calculated based on the standard curve.

An amylose/amylopectin kit (Megazyme, Bray, Ireland) was also used to assay 20–25 mg of biological sample from tubers for each event. The assay is based on the separation of amylopectin from amylose using Concanavalin A. This lectin binds and precipitates amylopectin, leaving amylose in the supernatant that can then be measured colorimetrically. Manufacturer’s instructions were followed for each step to prepare the samples, and then 40 µL aliquot from the final solution was transferred to a microtiter plate, and its absorbance was read at 510 nm on the SpectraMax 190 Plate Reader.

The statistical analysis was conducted using the software JMP Pro 15 [62], analysis of variance was conducted, Least Square means were calculated, and mean separation was based on Student’s *t*-test.

### 4.5. Starch Viscosity Measurements

Viscosity was measured using Rapid Visco Analyzer (RVA, PerkinElmer, Waltham, MA, USA), using freeze-dried samples. One gram of ground tuber flour sample was used for this assay. The moisture values for various samples were fairly consistent. Distilled water was added to one gram of each sample to bring final suspension weight to 28 g. Manufacturer’s instructions were followed to analyze each sample. The program used to calculate viscosity values was a standard program supplied by the equipment manufacturer for potato.

## Figures and Tables

**Table 1:** Specific gravity and percent dry matter values in wild-type (WT, Yukon Gold strain TXYG79) and six different knockout events. *n* = tubers from three plants, except in case of event T1-1 tubers from only two plants were available; different lower-case letters for each value in the same column indicate a significant difference between plant types; *p* ≤ 0.05 (Student’s *t*-test).

Line	Specific Gravity	Dry Matter (%)
WT	1.105 ab	26.0 abc
T1-1	1.094 abc	24.4 abc
T1-27	1.082 c	26.3 abc
T1-32	1.063 d	20.1 d
T2-2	1.109 a	27.1 a
T2-7	1.094 bc	23.5 c
T2-8	1.094 bc	24.5 bc

**Table 2:** Pasting properties of samples from wild-type (WT, Yukon Gold strain TXYG79) and gene edited events. Data represent mean values; *n* = tubers from three plants, except in case of event T1-1 tubers from only two plants were available; different lower-case letters for each value in the same column indicate a significant difference between plant types; *p* ≤ 0.05 (Student’s *t*-test with LS means).

Sample	Peak Viscosity (cP)	Trough Viscosity (cP)	Breakdown (cP)	Final Viscosity (cP)	Setback (cP)	Peak Time (min)	Pasting Temperature (°C)
WT	317.0 d	264.0 d	53.0 c	297.3 d	33.3 ab	5.8 d	67.0 c
T1-1	352.5 cd	297.5 cd	55.0 c	343.5 bcd	46.0 a	5.9 cd	66.5 c
T1-27	457.3 b	359.0 b	98.3 b	405.0 b	46.0 a	6.0 cd	68.1 bc
T1-32	307.7 d	265.0 d	42.7 c	324.7 cd	59.7 a	6.2 abc	67.0 c
T2-2	386.0 c	329.3 bc	56.7 c	380.7 bc	51.3 a	6.4 ab	70.2 a
T2-7	726.0 a	573.0 a	153.0 a	581.0 a	8.0 b	6.5 a	69.1 ab
T2-8	402.7 c	323.7 bc	79.0 bc	358.3 bc	34.7 ab	6.1 bcd	67.2 c

## Data Availability

Not applicable.

## Abbreviations

CRISPR/Cas9	Clustered regularly interspaced short palindromic repeats and CRISPR-associated protein 9
GBSS/*gbssI*	Granule-bound starch synthase
DSB	Double stranded break
NHEJ	Non-homologous end joining
INDELS	Insertions/Deletions
GFP/*gfp*	Green fluorescent protein
*nptII*	Neomycin phosphotransferase
WT	Wild-type
RVA	Rapid Visco Analyzer
EBN	Endosperm balance number
PV	Peak viscosity
cP	Centipoise
